# Daily work pressure and task performance: The moderating role of recovery and sleep

**DOI:** 10.3389/fpsyg.2022.857318

**Published:** 2022-07-28

**Authors:** Jørn Hetland, Arnold B. Bakker, Roar Espevik, Olav K. Olsen

**Affiliations:** ^1^Department of Psychosocial Science, Faculty of Psychology, University of Bergen, Bergen, Norway; ^2^Center of Excellence for Positive Organizational Psychology, Erasmus University Rotterdam, Rotterdam, Netherlands; ^3^Department of Industrial Psychology and People Management, University of Johannesburg, Johannesburg, South Africa; ^4^Department of Leadership and Command & Control, Swedish Defence University, Stockholm, Sweden

**Keywords:** recovery, performance, work pressure, longitudinal diary-study, sleep

## Abstract

Whereas previous research has focused on the link between (mental and physical) workload and task performance, less is known about the intervening mechanisms influencing this relationship. In the present study, we test the moderating roles of daily recovery and total sleep time in the relationship between work pressure and daily task performance. Using performance and recovery theories, we hypothesized that (a) work pressure relates positively to daily task performance, and that both (b) daily recovery in the form of psychological detachment and relaxation, and (c) total sleep time independently enhance this relationship. Our hypotheses were tested in a 30-day diary study with 110 officer cadets on a cross-Atlantic voyage on a Naval sail ship. The results of multilevel modeling lend support to all three hypotheses. Taken together, our findings suggest that recovery and sleep duration between shifts play a key role in the relationship between daily work pressure and task performance. We discuss the implications of these findings for the stressor-detachment model.

## Introduction

Work in high reliability organizations (HRO) can be very demanding. For example, naval flight operations in the military and computer operations in a nuclear power plant involve complex work activities in a high-risk environment, where individuals need to work in an almost error-free manner (Vogus et al., 2014). As an example from military operations, Bartone (2006) identified stressors like isolation, uncertainty, boredom, powerlessness, danger, and high workload as typical stressors soldiers face in their work. In such contexts, where severe unanticipated interaction of multiple failures is more likely to occur than in most other work environments–high-quality work performance is of paramount importance, particularly in periods of high peaks in demands and production (La Porte, 1996; Rochlin et al., 1998; Vogus et al., 2014). Hence, the possible detrimental effects of impaired task performance in these settings may be catastrophic, as witnessed for example in the Chernobyl nuclear reactor explosion in Ukraine in 1986 (LaPorte and Consolini, 1991).

High-quality work performance in these settings requires highly knowledgeable and technically skilled employees (Rochlin et al., 1998; Vogus et al., 2014). These employees must also be in optimal physical and mental states in order to obtain and maintain high levels of alertness, vigilance and situational awareness (Bakker, 2011), and thus able to fulfill their tasks effectively in dangerous situations, like during hurricanes or when confronted with an aggressive fire (Flin, 1996). Notably, several studies have identified recovery from work as an important mechanism behind employees’ cognitive and physical functioning over time, by revitalizing depleted cognitive and physical resources (Sonnentag and Fritz, 2015). Indeed, Shimazu et al. (2012) have shown that lack of rest and detachment from work (i.e., lack of recovery) coincides with reduced work engagement, psychological distress, and physical complaints. In the same vein, several studies have shown that lack of sleep, as a highly prevalent challenge in operational contexts (Miller et al., 2011), impairs performance on a wide range of cognitive, emotional, and technical tasks–and subsequently impairs task performance in professional settings by depleting personal resources (Harrison and Horne, 2000; Olsen et al., 2016). In contrast, recovery sleep after sleep deprivation shows restoring effects on these personal resources (Bonnet, 2011).

In their recent meta-analysis on employee recovery from work, Steed et al. (2021) point to the importance of both studying what people do to recover (e.g., recovery sleep) and their recovery experiences to capture a more comprehensive picture of the total recovery processes during both day and night. This may be especially important in HRO operational work-settings, where the quantitative workload and time pressure may fluctuate unexpectedly from very low to very high from one day to the next, and the consequences of impaired task performance can be devastating. Therefore, most HRO’s apply rigorous selection processes based on trait research in order to secure employees generally match the work challenges. However, studies show that even employees who generally perform well, may have off-days, which may be detrimental in a HRO setting. Thus, in addition to knowledge about *between-person* differences in explaining work performance, we need to expand knowledge regarding *within-person* differences. Within-person variations in exposure to work pressure and recovery may explain why individual performance fluctuates from one work-shift to the next (Beal et al., 2005). To examine daily fluctuations in work pressure and recovery, as well as performance, we designed a quantitative diary study encompassing 30 days of measurement in two samples of naval cadets on two 75-day training voyages across the North Sea and the Atlantic during the storm season on a 100-years old sail ship.

With this study, we aim to contribute to the literature in at least three ways. First, we contribute to the recovery literature by investigating the possible moderating roles of recovery experiences and total sleep time in the link between work pressure and task performance–on a daily basis. Do experience-based recovery strategies like psychological detachment and relaxation, and amount of sleep in-between shifts, strengthen the positive relationship between daily work pressure and performance? Whereas previous research has established relationships of daily work pressure with wellbeing (e.g., Sonnentag et al., 2010; Hetland et al., 2021), we focus on task performance in the current study. We use effort-recovery (Meijman and Mulder, 1998) and stressor-detachment (Sonnentag and Fritz, 2015) models to argue that effort expenditure at work is associated with acute load reactions (e.g., accelerated heart rate, fatigue) which demand compensatory effort in order to perform adequately at work. Specifically, we propose that recovery experiences and sleep can prevent an accumulation of strain and thus facilitate performance in response to daily work pressure. Second, we contribute to the work performance literature by providing important knowledge about what individual employees can do in order to optimize their task performance under high pressure. Third and finally, by conducting the study in an operational naval setting, our research also contributes to the field of operational psychology in HRO settings–adding knowledge about factors enhancing safety and operational performance. Thus, the present study may also provide novel insights relevant to the generalizability of evidence conducted in work settings where employees work under less constrained conditions like among knowledge workers and land-based industries.

### Work pressure and task performance

Task performance can be defined as the effectiveness with which an employee performs activities that contribute to the organization’s technical core, either directly by implementing a part of its technological process, or indirectly by providing it with needed materials or services (Borman and Motowidlo, 1993). For a naval officer, performing controlled navigation, leading combat or salvage operations, maneuvering a ship, and maintaining safety behavior are examples of such task behaviors, often corresponding to formal job descriptions. In the present study, we argue that naval cadets who are exposed to high levels of daily work pressure, will perform well–particularly if they have opportunities to recover between shifts.

According to the challenge-hindrance stressor model (Cavanaugh et al., 2000; LePine et al., 2005), there are two types of job demands, namely, hindrance and challenge job demands. Hindrance job demands refer to conditions and tasks that require effort and energy, but do not have much potential for learning and growth (LePine et al., 2005; Van den Broeck et al., 2010). Typical hindrance demands are role conflict, bureaucracy, and job insecurity, such job demands can actually thwart basic psychological needs (Coxen et al., 2021). In contrast, challenge job demands, including workload, task complexity, and deadlines present conditions and tasks that require effort and energy, but effectively dealing with them may result in growth, learning, and goal attainment. For example, short deadlines may require high levels of energy, but finishing tasks before the deadline can also promote mastery and competence.

Given that high work pressure on board of a sailing ship is often related to rapid handling of challenging and risky situations (e.g., a storm requiring hours of salvaging sails 40 m above deck at night), such work pressure may be perceived as an experience that motivates and stimulates performance, from day to day. Indeed, several previous diary studies have indicated that work pressure can act as a challenge job demand that is positively related to the willingness to invest considerable effort in work and perform well (e.g., Tadić et al., 2015; De Gieter et al., 2018). It should be noted that some diary studies did not find evidence for a direct positive relationship between work pressure and performance (e.g., Prem et al., 2018).

Work pressure may foster focus, involvement, and learning, and will challenge employees to use a variety of personal strengths and act proactively to reach their daily work goals. Approaching this issue from the opposite direction, Gardner (1986) argues that low levels of activation that may be due to a low work pressure, may cause apathy and low levels of performance. The idea here is that increases in work-related stimulation energize workers and their subsequent performance when the current stimulation level is low. At this backdrop, we also operationalize daily work pressure in the current study as work-pace demands and amount of work facing the officers (Van Veldhoven, 2014). Our quantitative diary study with repeated assessments allows us to control for an individual’s performance the previous day, and, consequently, examine to what extent daily work pressure is related to day-to-day *changes* in performance. We propose that daily work-pressure challenges naval cadets to invest all possible effort, which facilitates daily performance.

*Hypothesis 1:* Daily work pressure is positively related to daily task performance.

### The impact of recovery on the work pressure–performance relationship

The somewhat unclear association between work pressure and performance has led researchers to search for moderators of the relationship (de Jonge et al., 2012). Given the expected high expenditure of cognitive and physical resources when facing high work pressure in a HRO context like sailing a ship, we propose that the ability to regain personal resources, in terms of recovery, moderates this relationship. Recovery is a process during which individual functional systems that have been activated during a stressful experience return to their pre-stressor levels (Meijman and Mulder, 1998). This process can be viewed as the opposite of the strain process, encompassing both the ability to *refrain from* work demands (i.e., avoiding activities that call upon the same internal resources as those required at work), and *gain* new internal resources such as energy, self-efficacy, or positive mood, which may help to restore threatened resources (Sonnentag and Fritz, 2007).

Sonnentag and Fritz (2007) have argued that employees may engage in several off-job time *activities* to recover from work-related effort, but that recovery *experiences* are most important. The authors suggest four different recovery experiences, namely, psychological detachment, relaxation, mastery, and control. A review by Bennett et al. (2018) has shown that the first two strategies are most often investigated. *Detachment from work* refers to refraining from job-related activities and not thinking about work during non-work time. *Relaxation* implies low levels of mental or physical activation and little physical or intellectual effort. On a ship, mental distancing may take shape as planning of leisure activities at homecoming or daydreaming about family, and relaxation can be achieved during daily activities like training in the gym, watching movies, or reading books. However, such daily recovery experiences may be difficult to realize on a ship, where off-job time is spent on the workplace, physical space for leisure activities is sparse, and resting hours often disrupted by noise or even alarms involving all personnel. Therefore, it is likely that recovery will differ from day to day for individual officers. On the basis of previous studies, such lack in recovery is expected to impair task performance, particularly during high work pressure, due to sustained sympathetic activation (Meijman and Mulder, 1998). Impaired restoration of personal resources will mean that individuals have less energy and less cognitive capacity to carry out the work tasks (Binnewies et al., 2009; Hooff and Geurts, 2014).

Conversely, on days with low work pressure, high recovery may have a negative effect on performance, by adding more relaxation, and more resources into a state of already low activation, which may give rise to apathy and disengagement (Gardner, 1986). Furthermore, it can be expected that on days with low work pressure, a state of low recovery has less impact on performance, compared to a state of high recovery, because the body of resources needed to master work demands on such days are so low that performance will be maintained, despite lack of recovery. However, during days with high work pressure, such lack of recovery, and subsequently reduced personal resources, may lead to lower performance compared to high recovery states. Formally stated:

*Hypothesis 2a:* Daily psychological detachment moderates the positive relationship between daily work pressure and daily task performance. This relationship is stronger for those reporting high (vs. low) psychological detachment between work shifts.

*Hypothesis 2b:* Daily relaxation moderates the positive relationship between daily work pressure and daily task performance. This relationship is stronger for those reporting high (vs. low) psychological detachment between work shifts.

### The role of sleep

A central assumption in the present study is that recovery sleep and recovery experiences contribute to a person’s state of being recovered in a complementary way. The negative effects of sleep loss on performance are well documented in research covering a wide range of cognitive tasks and sensory functions, including skills relevant to effective functioning in an operational setting, like decision-making and planning (Harrison and Horne, 2000) as well as mood and positive affect (Dinges et al., 1997). According to Hobfoll (1989) an individual facing such resource depletion, particularly over time, will avoid challenges and transfer assignments to others in order to avoid investing further resources, and subsequently protecting a sense of mastery and self-image. This claim is supported for example by Olsen et al.’s (2016) finding that lack of daily sleep strongly impaired leadership performance in a naval bridge-team. Conversely, studies have shown that only one night of recovery sleep after even several days of sleep-deprivation has a strong and almost immediate positive impact on cognitive, technical, and physical performance, underscoring the restorative effect of sleep as part of the recovery process (Bonnet, 2011).

In sum, we suggest that it is likely that lack of daily sleep, particularly in situations with high workload and challenges, by depleting cognitive, affective, and motoric personal resources, may impair daily job performance (and vice versa). Hence, the relationship between work pressure and task performance may be moderated by total sleep time and sleepiness. As for recovery, we suggest that this relationship will be sensitive toward levels of work pressure. Thus, high job demands including work pressure may profit from longer sleep duration because sleep replenishes depleted resources, needed to master work tasks. In contrast, when the work pressure is relatively low, task performance may be less dependent of such restoration of resources and may even increase apathy and reduce performance due to a larger gap between energy levels and task demands (Gardner, 1986). It is also noteworthy that sleep differs greatly from night to night for the individual officers, due to factors like noise, alarms, and weather conditions (Nordmo et al., 2020). Notably, even though recovery processes contribute to replenishment of resources, we argue that sleep represents a supplementary strategy adding to the effects of physical and mental recovery, as defined in the current study. While detachment from work and relaxation can be considered as cognitive and emotional recovery processes obtained during waking time, sleep has additional and unique recovery characteristics in terms of biological recuperative processes that has been described in terms of several cellular mechanisms (e.g., Bonnet, 2011). Thus, we propose:

*Hypothesis 3:* Total sleep time moderates the positive relationship between daily work pressure and daily task performance. This relationship is stronger for those reporting high (vs. low) total sleep time between shifts.

## Materials and methods

### Participants and procedure

A total of 115 Norwegian naval cadets from a Military University College participated in our study. As part of their leadership training, the cadets traveled across the North Sea and the Atlantic from northern Europe to North America by sail ship. The data for the present study were collected during two voyages that took place in the fall of 2010 and the fall of 2011. All cadets participating on the two voyages were invited, and all the invited cadets volunteered to take part in the study. As an incentive to participate the cadets were promised to receive an individual report based on the daily measurements to be used for their personal development when they returned from the voyage. The invited participants were informed about the objective of the study and gave their written consent to participate 2 or 3 days before leaving port. In the written consent, it was clearly stated that participation was totally voluntary and that the cadets were free to withdraw from the study at any point in time without providing any reason. During the voyage the cadets participate in several different work tasks like sailing maneuvers, safety drills, and maintenance of the ship. Both in 2010 and 2011, data were collected using two different questionnaires. First, prior to the voyages, participants responded to a general survey measuring individual differences including demographics and participants’ general task performance. Secondly, the participants received a booklet with diary questionnaires for the first 30 days of their voyage including questions assessing work pressure, task performance, psychological detachment, relaxation, and total sleep time. We requested the cadets to fill out the questionnaire at 5 p.m. on each day. The initial sample of the first data collection consisted of 54 cadets, while the initial sample of the second data collection consisted of 61 cadets. In the initial sample of the second data collection, five of the participants did not respond to the general questionnaire and were therefore excluded from the final sample. The final sample, combining the two data collections, consisted of 95 male cadets (86.4%) and 15 female cadets (13.6%) making up a total of 110 participants. The mean age of the participants was 23.46 years (*SD* = 2.96), and their age ranged from 19 to 33. Of the 110 cadets participating in the study, 98 were from the naval branch, 11 from the armed forces, and 1 from the air force. Out of the possible 3300 measurement occasions we obtained 2381 responses, yielding a response rate of 72.15% across the 30 days.

### Measures

All measures used in the present study were based on existing scales translated to Norwegian using translation and back-translation (Brislin, 1970).

#### Trait survey

In order to measure general task performance we used four items from the task performance subscale developed by Goodman and Svyantek (1999). The participants were asked to respond to the following statements about their usual performance when they are on duty. Example items are: “I achieve the objectives of my job,” and “I fulfill all the requirements of my job.” The respondents were asked to respond on a five-point scale ranging from totally disagree (1) to totally agree (5). The scale showed reasonable reliability (Cronbach’s α = 0.74).

#### Daily diary booklet

We used daily diaries to measure fluctuations in our study variables. All day-level questionnaires were adapted versions of existing scales. We adapted the time frame of the scales and the number of items so the questions could be answered on a daily basis (cf. Ohly et al., 2010). Moreover, some of the introductory text and items were also adjusted to fit the military context on board of the sail ship.

**Day-level work pressure** was measured with four items referring to quantitative and time pressuring aspects of work. Items were based on a scale developed by Van Veldhoven et al. (2002). The items are “Today, I have had to work extra hard in order to complete something,” “Today, I had to work fast,” “Today, I had too much work to do,” and “Today, I have been working under time constraints.” Responses were given on a five-point frequency scale, ranging from not at all (1) to a very large degree (5). The average within-level reliability coefficient (Cronbach’s alpha) was 0.91, and reliability coefficients were in the range from 0.82 to 0.95 across the 30 days. The Norwegian version of the scale have been used three previous studies functioning as theoretically expected (Bakker et al., 2020; Ågotnes et al., 2021; Hetland et al., 2021).

**Day-level recovery** was measured with six items from the Recovery Experience Questionnaire (Sonnentag and Fritz, 2007) where three of the items were from the psychological detachment subscale and three from the relaxation subscale. The items were following a headline stating “When I have not been on duty the last twenty-four hours…” Example items are “I have distanced myself from my work” (psychological detachment), and “I have done relaxing things” (relaxation). We used the same answer format as for task performance. The internal consistencies for the two subscales were tested across all days. The average within-level reliability coefficient (Cronbach’s alpha) for the psychological detachment subscale was 0.75, and reliability coefficients were in the range from 0.56 to 0.86 across the 30 days. For the relaxation subscale the average within-level reliability coefficient (Cronbach’s alpha) was 0.89, and reliability coefficients were in the range from 0.77 to 0.94 across the 30 days. The Norwegian version of the scales has shown to be functioning as theoretically expected in a previous study (Hetland et al., 2021).

**Total sleep time** was measured by one single item. The item was phrased in the following way: “How many hours have you been sleeping the last twenty-four hours?” followed by a line where to enter the number of hours.

**Day-level task performance** was assessed with four adjusted items from the task performance subscale developed by Goodman and Svyantek (1999). Example items are “Today, I have achieved the objectives of my job”; “Today, I have fulfilled all the requirements of my job.” Responses were given on a five-point frequency scale, ranging from totally disagree (1) to totally agree (5). The average within-level reliability coefficient (Cronbach’s alpha) was 0.89, and reliability coefficients were in the range from 0.75 to 0.97 across the 30 days. The Norwegian version of the scale has shown to be functioning as theoretically expected in three previous studies (Bakker et al., 2020, 2022; Sørlie et al., 2022).

### Strategy of analysis

In order to capture the multilevel structure of the data, implying that the 30 daily measurements (level 1) of the study constructs were nested within individuals (level 2), we applied multilevel analyses by the use of MLwiN 3.05. In the analyses, the level 1 (day level) predictors were centered on the respective person mean, while the level 2 (person level) variable was centered on the grand mean. In the multilevel analyses, we tested four models in the prediction of task performance. First an unpredicted null model (model 1) was tested, followed by a predicted main effect model (model 2), and two interaction models (Model 3 and 4). In all of the predicted models, we included the respondents general task performance and the average score of all predictors on the person-level following the procedure suggested by Bolger and Laurenceau (2013, pp. 77–78). In main effect model, we added the day-level predictors (daily work pressure, psychological detachment, relaxation, and total sleep time). In the first of the two interaction models (Model 3), the three hypothesized interactions between work pressure and the three predictors were added to the main effect model, while in the second interaction model (Model 4) we also included previous-day task performance. By controlling for the uncentered levels of previous-day task performance in the second interaction model, the temporal stability in the construct is accounted for. Hence, the explained variance in daily task performance can be interpreted as a positive or negative change in task performance from the previous to current day. In contrast, the relationship in the model not controlling for previous-day task performance should be regarded as a simultaneous (cross-sectional) effect.

#### Pre-analysis

To rule out the potential danger of conceptual overlap between the daily measurement of work pressure and work performance, we conducted a multilevel confirmatory factor analysis using their respective observed indicators across the 30 days. Testing the expected correlated two-factor solution resulted in a good overall fit to the data (χ^2^ = 219.85, DF = 38, CFI = 0.98, TLI = 0.98, and RMSEA = 0.04), as well as a good fit on the two specific levels (SRMR within = 0.02 and SRMR between = 0.03). On the within-level, factor loadings were in the range of 0.66–0.89, and on the between-level in the range of 0.85–0.98. The two constructs correlated positively on the within-level (*r* = 0.10, *p* < 0.001) and negatively on the between-level (*r* = −0.28, *p* < 0.001). Hence, the multilevel factor analysis supported the validity of considering the two concepts as two separate constructs on both levels.

## Results

### Descriptive statistics

Means, standard deviations, within person- and between person-level correlations for all study variables are presented in Table 1. A significant, but weak positive correlation was found between daily work pressure and task performance the same day at the within-level (*r* = 0.08, *p* < 0.001). Moreover, a significant negative association was found between psychological detachment and task performance (*r* = −0.07, *p* < 0.001), and between total sleep time and task performance (*r* = −0.04, *p* = 0.034). Daily relaxation was not related to task performance at the within-level (*r* = −0.03, *p* = 0.101).

**TABLE 1:** Means, standard deviation, and within person- and between person level correlations for study variables (*N* = 3300 occasions, *N* = 110 participants).

Variables	$\bar{\text{x}}$	*SD*	1.	2.	3.	4.	5.	6.
**Day-level**								
1. Task performance	3.96	0.58		0.08*	–0.07**	–0.03	–0.04*	–
2. Work pressure	2.50	0.88	–0.26*		–0.19**	–0.26**	-0.15**	–
3. Psychological detachment	2.67	0.87	0.00	–0.16		0.31**	0.11**	–
4. Relaxation	2.73	1.03	0.20	–0.26*	0.37**		0.18**	–
5. Total sleep time	6.09	1.25	0.15	–0.12	0.12	0.32**		–
**Person-level**								
6. General task performance	4.23	0.46	0.52**	–0.02	–0.04	0.19	0.05	

Correlations below the diagonal are correlations on the between (person) level and correlations above the diagonal are correlations on the within (day) level.

*p < 0.05, **p < 0.001.

### Multilevel analysis

Table 2 presents the results from the multilevel analysis predicting daily task performance. Prior to testing the predicted models, an unpredicted model (null-model) should be tested to confirm that there is sufficient day-level variance in the current dependent variable. As shown in Table 2, the initial unpredicted model revealed significant variation in daily task performance on both the day-level (70.6%) and person-level (29.4%) allowing us to proceed with testing the hypothesized multilevel models.

**TABLE 2:** Multilevel analysis.

	Null model		Main effect model		Interaction model 1 (Simultaneous-effect)		Interaction model 2 (Change-effect)	
				
	*B*	*SE*	*B*	*SE*	*B*	*SE*	*B*	*SE*
Intercept	3.955**	0.032	3.953**	0.027	3.965**	0.027	3.971**	0.027
General task performance			0.352**	0.060	0.356**	0.060	0.359**	0.061
Average work pressure (person level)			–0.131**	0.051	–0.125*	0.051	–0.113*	0.051
Average psychological detachment (person level)			–0.021	0.048	–0.019	0.048	–0.023	0.048
Average relaxation (person level)			0.014	0.054	0.009	0.055	0.007	0.055
Average total sleep time (person level)			0.039	0.047	0.044	0.048	0.035	0.048
Work pressure			0.051**	0.014	0.060**	0.014	0.053**	0.014
Psychological detachment			–0.042**	0.016	–0.042**	0.016	–0.038*	0.016
Relaxation			0.006	0.012	0.009	0.012	0.031**	0.012
Total sleep time			–0.012	0.009	–0.012	0.009	–0.005	0.009
Work load × Psychological detachment					0.034	0.021	0.044*	0.022
Work load × Relaxation					0.041**	0.016	0.013	0.017
Work load × Total sleep time					0.035**	0.011	0.028*	0.012
Task performance previous day							0.184**	0.020
Variance level 2 (person level)	0.099	0.015	0.067	0.010	0.069	0.011	0.069	0.011
Variance level 1 (day level)	0.238	0.07	0.237	0.007	0.234	0.007	0.210	0.006
-2 Log likelihood	4131.764		3978.732		3947.936		3261.274	

Daily task performance by work pressure, psychological detachment, relaxation, and total sleep time controlled for general task performance and average person-level scores for all predictors (*N* = 3300 occasions, *N* = 110 participants).

**p < 0.01, *p < 0.05.

In hypothesis 1, we propose a positive relationship between work pressure and daily task performance. As can be seen in Table 2, in the main effect model a positive relationship was found between daily work pressure and daily task performance (*B* = 0.051, *p* < 0.001) after controlling for general task performance and the average scores for all predictors at the person-level. Hence, hypothesis 1 was supported. Moreover, the main effect model also revealed a significant negative relationship between daily psychological detachment and daily task performance (*B* = −0.042, *p* < 0.001), while no significant main effects were found for either relaxation, or sleep duration.

**FIGURE 1:**
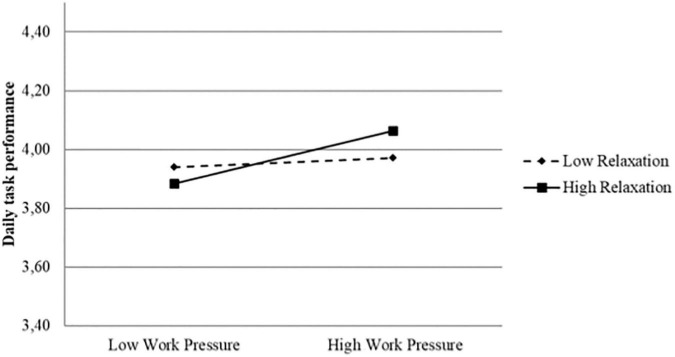
Significant interaction effect between daily work pressure and daily relaxation on daily task performance (Simultaneous-effect model).

In hypothesis 2a and 2b, we predict that daily psychological detachment and relaxation positively moderate the relationship between work pressure and daily task performance. As shown in Table 2, the first interaction model investigating simultaneous effects (Model 3), revealed a significant interaction between work pressure and relaxation (*B* = 041, *p* < 0.001), while the hypothesized interaction effect between work pressure and psychological detachment was not significant (*B* = 0.034, *p* = 0.053). The work pressure × relaxation interaction effect is illustrated in Figure 1. The figure indicates higher task performance on days with higher work pressure among naval cadets who reported high relaxation between shifts, while task performance is unrelated to work pressure among those who reported low relaxation. To formally test the significance of the slopes in the interaction, we conducted a simple slope test at the condition of ±1 SD for both the predictor and moderator. The results revealed a significant positive slope for those reporting high relaxation (Slope = 0.102, *z* = 4.649, *p* < 0.001), and a non-significant slope for those scoring low in relaxation (Slope = 0.018, *z* = −0.798, *p* = 0.425). In sum, the results from the interaction model investigating simultaneous effects supported hypothesis 2b, while hypothesis 2a was not supported in the simultaneous-effect model.

**FIGURE 2:**
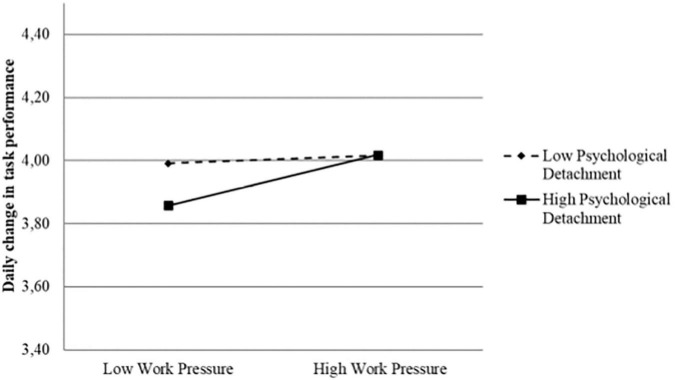
Significant interaction effect between daily work pressure and daily psychological detachment on daily change in task performance (Change-effect model).

In the second interaction model (Model 4), we controlled for previous day’s levels of task performance in addition to the main effects and interaction effects included in simultaneous effect model. Hence, a positive relationship represents an increase in task performance from the previous day to the current day, and a negative relationship would represent a respective decrease in task performance. As shown in Table 2, the work pressure × relaxation interaction effect was not significant (*B* = 0.013, *p* = 0.222) when predicting *change* in daily task performance, whereas now the work pressure × psychological detachment interaction became significant (*B* = 0.044, *p* = 0.023). In accordance with the hypothesized enhancing effect of psychological detachment in hypothesis 2a, Figure 2 indicates a clear positive relationship between work pressure and daily changes in task performance among cadets reporting high psychological detachment, while the corresponding relationship among those reporting low psychological detachment was almost flat. Noteworthy, cadets who reported high psychological detachment between shifts showed lower daily task performance compared to cadets scoring low psychological detachment when work pressure was low. Consistent with Figure 2, formal testing of the slopes revealed a significant positive slope for those reporting high psychological detachment (Slope = 0.091, *z* = 3.71, *p* < 0.001), and a non-significant slope for those reporting low psychological detachment (Slope = 0.015, *z* = 0.269, *p* = 0.529). Hence, the second interaction model predicting *change* in daily task performance supported hypothesis 2a, while hypothesis 2b was not supported in the model.

**FIGURE 3:**
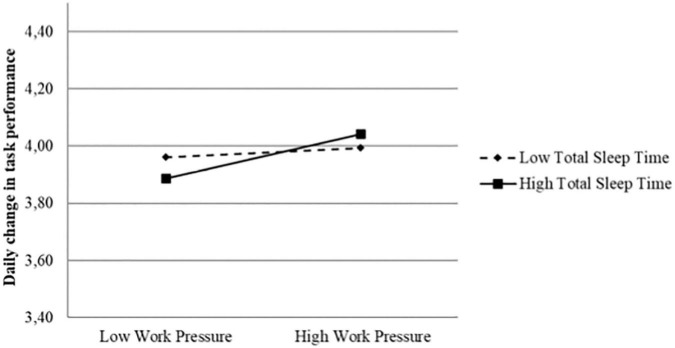
Significant interaction effect between daily work pressure and total sleep time on daily change in task performance (Change-effect model).

In hypothesis 3, we propose that sleep time during the previous night positively moderates the relationship between daily work pressure and daily task performance. As can be seen in Table 2, the work pressure × total sleep time interaction effect was significant in both interaction models (*B* = 0.035, *p* < 0.001 and *B* = 0.28, *p* = 0.010, in the simultaneous-effect model and change-effect model, respectively). The interaction effect from the change-effect model is illustrated in Figure 3. As can be seen, work pressure resulted in positive changes in task performance for individuals who reported high total sleep time, while there was no such effect for individuals who reported low total sleep time. Notably, cadets reporting high total sleep time showed lower daily task performance compared to cadets reporting low total sleep time when work pressure was low. In line with predictions, formal testing of the slopes at the conditions of ±1 SD for both the predictor and moderator revealed a significant positive slope for those with many sleep hours in-between the shifts (Slope = 0.088, *z* = 4009, *p* < 0.001), while the slope for those with limited number of sleeping hours was non-significant (slope = 0.018, *z* = 0.918, *p* = 0.359). The pattern and slopes of the interaction effect revealed in the simultaneous-effect model was almost identical to the interaction revealed in the change-effect model. Hence, hypothesis 3 was supported in both interaction models.

## Discussion

The present study tested the link between work pressure and day-to-day changes in task performance in an operational naval setting. The central aim was to investigate whether daily recovery experiences and sleep duration would qualify this relationship. The results provide evidence for a direct link between daily work pressure and daily task performance, supporting that work pressure generally acts as a challenge demand for all individuals. Moreover, as hypothesized, the findings revealed that recovery experiences and total sleep time in-between shifts positively moderated the work pressure–performance relationship. Nevertheless, some temporal differences were found in the functioning of relaxation and psychological detachment in the work pressure–performance relationship. Work pressure was positively related to simultaneous levels of task performance on the days that were preceded by relaxation, while work pressure was related to positive changes in task performance on the days preceded by psychological detachment. In what follows, we discuss the most important theoretical and practical contributions of the study.

### Theoretical contributions

Individuals who work in HRO are often exposed to high job demands, but are expected to work in an error-free manner (Vogus et al., 2014). Particularly on the days that there are high peaks in demands–for example, when confronted with extreme weather conditions, high work pressure and intense training–high-quality work performance is crucial (La Porte, 1996; Rochlin et al., 1998; Vogus et al., 2014). In the present study, daily work pressure was positively related to job performance, but this relationship was relatively weak. Moreover, when controlling for previous-day performance, the work pressure–performance relationship disappeared. These findings indicate that work pressure was *not* a positive predictor of performance, which is inconsistent with the challenge-hindrance stressor framework (Cavanaugh et al., 2000; LePine et al., 2005). According to this framework, hindrance job demands such as role ambiguity and interpersonal conflicts present conditions that require effort and energy, but do not have growth potential and do not contribute to performance (LePine et al., 2005; Crawford et al., 2010). In contrast, challenge demands, including work pressure and job complexity present work tasks and conditions that require effort and energy, but efficient dealing with them results in growth, mastery, and goal attainment. It should be noted that Prem et al. (2018) in a previous diary study also failed to demonstrate the expected positive link between time pressure and performance on the within-level among knowledge workers in diverse occupations and industries. Hence, this suggests that the findings from the present study may provide useful insights that also generalize to more mundane contexts than the constrained HRO setting being the focus of the present study.

One possible explanation for the null finding in the present study may be that the work pressure was simultaneously motivating and exhausting for the naval cadets–together resulting in a null effect on performance. Importantly, most previous studies on the hindrance-challenge stressor framework have used cross-sectional or longitudinal research designs–testing differences in wellbeing (which would help to perform well) *between* individuals exposed to low versus high levels of work pressure. The present study shows that when the focus shifts to *within-person* effects–i.e., on variations in work pressure from day to day, work pressure may actually not contribute to explaining wellbeing and goal attainment (see also Breevaart and Bakker, 2018).

Another explanation for the null findings offered by the current study is that not all cadets recovered sufficiently in-between the shifts to be able to respond to work pressure with excellent performance. According to both the effort-recovery model (Meijman and Mulder, 1998) and the stressor-detachment model (Sonnentag and Fritz, 2015), recovery experiences can buffer the link between job demands and strain. The model proposes that psychological detachment from work offers opportunities to recover from work-related stress and build new physical and psychological resources (e.g., vitality). Such resources can then be used to perform well, and particularly on days with high work pressure. Hetland et al. (2021) expanded this model by proposing and empirically investigating that relaxation may similarly offer opportunities for recovery, so that employees have sufficient energy to optimize their performances on challenging days. Although their findings only supported the job demands × recovery interaction for psychological detachment, the current study found evidence for a boosting role of relaxation as well as sleep duration. Thus, cadets who relaxed and slept well in-between shifts were better able to transform work pressure into adequate performance. These findings expand the stressor-detachment model by showing that next to psychological detachment it is relaxation and sleep duration that presumably restore the energy reservoir so that employees can perform well on busy days.

Nevertheless, one interesting finding is that relaxation only boosts the work pressure-task performance relationship when looking at simultaneous effects within the same day, while the boosting role of psychological detachment only occurred when predicting changes in task performance from one day to another. A possible explanation to this is that detachment relates more explicitly to what happened yesterday (e.g., not thinking about work when off duty) than relaxation. The individual distances themselves psychologically from the events that happened at work, including all events that affected performance. This works particularly well when analyzing changes in job performance. On the days individuals realize an increase in their performance, they have to invest additional effort, which is more likely after shifts during which cadets have detached from previous-day’s events that influenced previous-days’ performance. When cadets detach from their work-related efforts, they can build new energy resources by engaging in leisure time activities such as studying, exercising, and socializing. These new resources can be used during the next day to deal with work pressure and perform well.

It should be noted that an interesting sub-finding in the present study is a negative relationship between psychological detachment and daily task performance. One possible explanation to this unexpected negative relationship could be that when individuals detach themselves psychologically from their work, they no longer invest energy in the work activity. In contrast, when still thinking about work during off-job time, people may solve problems that may facilitate performance in the short run. Indeed, Niks et al. (2017) found that employees who did not detach from work during leisure time were more creative.

A last noteworthy finding in the present study was that the items supposed to measure psychological detachment evoked inconsistent responses, resulting in an unreliable measure during four of the 30 days of study (in 13% of the cases). Although it is not unusual to see fluctuations in reliabilities across days in diary studies, these findings suggest that distancing from work was not perceived similarly across days. We can only speculate about the reasons for this, and this may be fruitful for future research in which scholars try to explain inconsistencies in their daily assessments. For example, it is conceivable that detachment from work is more difficult and psychologically different if one tries to recover on the work location as compared to home or another off-job location. Of course, the cadets who participated in the present study were each day requested to report the extent to which they could psychologically detach from work. Perhaps detachment was almost impossible on some of the days (e.g., days with stormy weather), making the concept erroneous during these days. The lesson for diary research is that the phenomenology of the concepts studied may fluctuate and be a function of the environment in which these concepts are studied.

### Limitations and future research

The present study has several limitations that should be taken into account when interpreting the findings. First, we used self-reports, which may have led to common method variance–which increases the risk of inflated correlations. However, the correlations in the present study were generally low, which speaks against inflated correlations. Still, future research may want to include other-ratings of job performance and a separation in time of the independent and dependent variables. Second, applying booklets usually gives researchers no or very limited control over when participants fill in the survey. It might be that some participants filled in several surveys at the same time. It might be that they responded to the surveys at bedtime rather than in the afternoon. However, in order to make sure that participants filled in the daily questionnaires at the correct time (each day at 5 p.m.), we asked squad leaders to motivate the cadets to fill out the booklets at the right time. Also, the researcher on board on the ship saw to it that the right procedure was followed. It should also be noted that at the time of the study, we did not have an alternative to booklets, because in the mid of the North Sea and the Atlantic Ocean cadets have no access to the internet.

Third, our sample of naval cadets was rather specific, and can of course not be taken as representative of the general working population. Still, the findings were generally in line with stressor-detachment and effort-recovery models, which have mostly been studied among white-collar, office workers. Moreover, our findings do have relevant implications for studying task performance in office workers. Our findings show that employee job performance changes as a function of fluctuations in work pressure (from very low to very high), and that recovery is crucial for a positive linkage between work pressure and performance. Previous studies among employees working in a range of occupations (e.g., Tadić et al., 2015; De Gieter et al., 2018) have indicated that such fluctuations can be found in most sectors, and that therefore psychological detachment, relaxation, and sleep most likely have a similar restorative function in other occupational groups. Nevertheless, as discussed above, the working conditions on board of the sail ship are rather unique in that the participants spend most of their time together during an extended period of time. Moreover, off-job time is spent on the same location as work, which most likely increases the risk of work-to-non-work interference and decreases opportunities for distancing from work. Future research should therefore try to replicate the current findings in other occupational settings. Finally, the sample used in the present study was young (23 years), and most participants were male. Research has shown that with increasing age, individuals lose and gain personal resources (e.g., decreased fluid intelligence, increased crystallized intelligence, decreased physical health, increased wisdom and experience; Truxillo et al., 2015)–which may all affect how individuals deal with and respond to job demands. It would seem important and interesting to test age effects in the stressor-detachment model in future research.

### Practical implications

The findings from the present study have several practical implications. First, in order to optimize employee job performance on days with high work pressure, organizations and their leaders should assure that employees have sufficient opportunities to recover and actually engage in recovery activities during their off-job time. This is especially important in a work setting where employees both work and recover within the same physical environment like on board of an oil-tanker, off-shore platform or during military operations on board of a ship. Hence, in such work settings organizations should assure that employees have appropriate conditions to disengage from work and relax. This could for example be done by providing access to a sufficient number of quiet rooms ideal for reading a book, using a tablet, and watching a movie (using head phones) or a gym and swimming pool where employees can exercise, relax, and socialize.

Second, the knowledge from the present study could also be used by HR departments and consultancy firms to develop training programs to improve and facilitate employees’ recovery experiences and activities. We have shown that recovery experiences as well as sleep have the potential to facilitate employee functioning when confronted with high levels of daily work pressure. Trainers may use this knowledge to develop tools and exercises through which employees learn how, when, and where to detach from work and relax–by engaging in certain activities during leisure time (e.g., socializing, reading, exercise) and abstaining from other activities (e.g., using smartphones just before bedtime). Finally, the findings from the present study show that total sleep time has an additional and complementary boosting effect on the link between the effort invested by the employees and their performance. Hence, organizations and their leaders should also assure good facilities and conditions for employees’ recovery sleep. They may distribute educative apps and video material developed to facilitate falling to sleep and maintaining consistent sleep through the whole sleeping period.

## Conclusion

The present study shows that psychological detachment, relaxation, and sufficient sleep help dealing with daily job demands in the form of work pressure. Individuals who are able to detach from their work, relax during their leisure time and who get enough sleep seem able to change high work pressure into a challenge job demand that helps them to perform well. Since previous research has shown that work pressure is an important predictor of job stress that undermines performance (e.g., Taris, 2006), the current study shows that daily recovery is crucial for effective coping with daily job demands and efficacious daily job performance.

## Data availability statement

The raw data supporting the conclusions of this article will be made available by the corresponding author on resonable request.

## Ethics statement

Ethical review and approval was not required for the study on human participants in accordance with the local legislation and institutional requirements. The patients/participants provided their written informed consent to participate in this study.

## Author contributions

JH organized the database and performed the statistical analysis. JH, AB, and OO were responsible for writing the first draft of the manuscript. All authors contributed to study design, data collection, and manuscript revision, read, and approved the submitted version.
